# Patients’ Lived Experience in a Multicomponent Intervention for Fibromyalgia Syndrome in Primary Care: A Qualitative Interview Study

**DOI:** 10.3390/ijerph192013322

**Published:** 2022-10-15

**Authors:** Victoria Mailen Arfuch, Rosa Caballol Angelats, Carina Aguilar Martín, Alessandra Queiroga Gonçalves, Noèlia Carrasco-Querol, Gemma González Serra, Maria Cinta Sancho Sol, Immaculada Fusté Anguera, Emilie Friberg, Anna Berenguera

**Affiliations:** 1 Unitat de Suport a la Recerca Terres de l’Ebre, Fundació Institut Universitari per a la Recerca a l’Atenció Primària de Salut Jordi Gol I Gurina (IDIAPJGol), 20 Cristòfol Colom Avenue, 43500 Tortosa, Spain; 2 Department of Pediatrics, Obstetrics and Gynecology and Preventive Medicine and Public Health Universitat Autònoma de Barcelona, 08193 Bellaterra, Spain; 3 Division of Insurance Medicine, Department of Clinical Neuroscience, Karolinska Institutet, 3 Berzelius väg Street, 6 Floor, 171 77 Stockholm, Sweden; 4 Centre d’Atenció Primària (CAP) El Temple, Gerència Territorial de Terres de l’Ebre, Institut Català de la Salut (ICS), Plaça Carrilet sn., 43500 Tortosa, Spain; 5 Unitat d’Expertesa en Sindromes de Sensibilització Central Terres de l’Ebre, Gerència Territorial de Terres de l’Ebre, Institut Català de la Salut (ICS), 20 Cristòfol Colom Avenue, 43500 Tortosa, Spain; 6 Unitat d’Avaluació, Direcció d’Atenció Primària Terres de l’Ebre, Gerència Territorial de Terres de l’Ebre, Institut Català de la Salut (ICS), 20 Cristòfol Colom Avenue, 43500 Tortosa, Spain; 7 Unitat Docent de Medicina de Família i Comunitària Tortosa-Terres de L‘Ebre, Institut Català de la Salut (ICS), 44-58 Esplanetes Street, 43500 Tortosa, Spain; 8 Servei de Rehabilitació i Medicina Física, Hospital de Tortosa Verge de la Cinta, Gerència Territorial de Terres de l’Ebre, Institut Català de la Salut (ICS), 44-58 Esplanetes Street, 43500 Tortosa, Spain; 9 Centre de Salut Mental d’Adults (CSMA) de Fundació Pere Mata Terres de l’Ebre, 50 Rambla de Pompeu Fabra, 43500 Tortosa, Spain; 10 Central Research Unit, Fundació Institut Universitari per a la Recerca a l’Atenció Primària de Salut Jordi Gol I Gurina (IDIAPJGol), 08007 Barcelona, Spain; 11 Department of Nursing, Universitat de Girona, Plaça de Sant Domènec, 3, 17004 Girona, Spain

**Keywords:** fibromyalgia syndrome, primary care, multicomponent intervention, qualitative research, interview study, thematic analysis

## Abstract

Fibromyalgia syndrome (FMS) disrupts patients’ biopsychosocial spheres. A multicomponent intervention (MCI) program, which combined health education, cognitive behavioral therapy, and physical activity, was conducted in South Catalonia’s primary care centers with the aim of improving symptom self-management and quality of life. A qualitative interview study was carried out to understand patients’ lived experiences during the intervention program. Sampled purposively, 10 patients were interviewed via phone calls and face-to-face. The encounters were audio-recorded, verbatim transcribed, and analyzed through thematic analysis. As a result, four themes emerged: legitimizing fibromyalgia through the MCI, the MCI as a socializing experience, learning how to live with FMS through the MCI, and room for improving the MCI. Participants agreed on the program being an insightful experience that promoted illness knowledge and acceptance and that improved their coping skills and symptom self-management. The inclusion of additional psychological guidance, expressive psychological group therapy, and providing relatives with information were proposed for enhancing the program. Our findings have contributed to gaining insight into the subjective impact of the MCI and identifying new therapeutic targets to tailor the program to patients’ needs, which will hopefully increase its effectiveness and improve their quality of life.

## 1. Introduction

Fibromyalgia syndrome (FMS) is a chronic condition that impacts patients in multiple directions. It compromises their personal, social, working performance, and quality of life (QOL) and potentially leads to mental health problems and disability [1,2]. Its complexity is given by the similarity of its symptoms with other diagnoses, the variability of the clinical picture, the associated comorbidities, and its patients’ profiles [3]. FMS is mainly characterized by widespread musculoskeletal pain and fatigue, yet its etiopathogenesis and gold standard treatment options are under-researched and carry ethical and scientific controversies [4].

The burden on society seems undeniable in terms of healthcare services and productivity losses [5]. Being classified as a central sensitivity syndrome [6,7], FMS is the most frequent among rheumatic diseases, with a worldwide prevalence of 2.7% and a prevalence of 2.4% in Spain [8,9]. FMS is more common among women, as well as among those over 50 years of age; with low levels of education; with low socioeconomic statuses; living in rural areas; and possibly, who are overweight [10]. Even though the reported sex difference is still not medically understood, studies propose a diagnosis criterion bias [11,12,13]. In line with this, Valls [14] suggests a gender bias in searching for a differential diagnosis, while Martínez-Lavín [15] proposes a different approach considering fibromyalgia as a sex-dimorphic neuropathic pain syndrome frequently observed in women. Additionally, the literature also stresses the resemblance of FMS with perimenopause and menopause symptoms and its potential link with a hormone deficit-related disorder [16,17].

Existing research indicates that poor perception of illness, including perceiving a fluctuating and unpredictable illness course and a low emotional representation of FMS, which means not connecting emotionally with one’s health condition, is associated with higher medical costs [18]. Therefore, healthcare approaches may benefit from including patients’ subjective experiences of living with and being treated for FMS.

In the literature on FMS illness experience, the most prevalent themes include the inauthenticity of the syndrome and the uncertainty about its prognosis, identity and family disruptions, distress, social stigma, pain management, illness acceptance, and alternative healthcare approaches [19,20,21,22,23,24].

While there has been a great deal of research on FMS patients’ illness narratives, to the best of our knowledge, very few studies have been designed to assess FMS interventions implementing the qualitative methodology [25,26]. Relevant but limited, the evaluation of intervention programs and healthcare technologies is commonly based on quantitative techniques to measure their effectiveness on health status, symptomatology, and functionality but not on patients’ accounts about their perception of change and benefits. Exploring patients’ subjective treatment experiences will provide novel insights into patients’ health needs and a broader understanding of the barriers and facilitators of a new healthcare strategy to be tailored according to these results [21,22,23,24,25,26,27]. Furthermore, this approach is in correspondence with the mix-method strategy recommended by the Medical Research Council guidance when evaluating complex interventions [28].

According to international guidelines and reviews, multicomponent interventions (MCI) based on physical activity, cognitive behavioral therapy (CBT), health education, and pharmacotherapy are beneficial to addressing FMS [4,29,30]. Additionally, published evidence suggests a promising future for this multimodal therapeutic approach [25,26,31,32,33,34,35].

Initiatives for delivering interdisciplinary healthcare in Catalonia’s primary care sector (Spain) have been operating since 2016 [36]. Currently, an ongoing MCI project is being run by the Central Sensitivity Syndromes Unit in the Gerència Territorial Terres de L’Ebre (GTTE) of the Institut Català de la Salut (ICS) [37]. This MCI program seeks to strengthen routine practice by providing non-pharmacological resources for symptomatic control and biopsychosocial suffering relief including health education, physical activity, and CBT. Nevertheless, MCI programs are still in their onset in the Spanish public health system and elsewhere globally, so there is scope for further research.

Our study aims to understand the structure and meanings of patients’ lived experiences during the proposed MCI program and to explore its subjective insight. Specifically, this study plans to identify informers’ overall perception of benefits from the MCI; to detect the extent to which the informers implement the coping strategies delivered during the program in their daily lives; and to explore to what degree this experience may have influenced informers’ psychological, physical, and social spheres and contributed to personal growth. Additionally, we aim to detect improvement aspects of the program according to participants’ views.

The results will be helpful to adapt the intervention according to patients’ appraisals in order to reinforce its benefits. Furthermore, this study is expected to complement the quantitative results of the randomized controlled trial (RCT) linked to this MCI (ClinicalTrials.gov: NCT04049006) [37] and the recently published findings of a focus group study conducted on the same project [38].

## 2. Materials and Methods

### 2.1. Design

The design of this work involves a qualitative interview study through thematic analysis [39].

### 2.2. Multicomponent Intervention Program and Setting

The MCI program includes a 12-week/2-h session group-based interdisciplinary program in addition to the usual clinical care (UCC). The latest is delivered cost-free by the public health system and is generally based on the screening of a general practitioner, the delivery of general information about the syndrome and coping strategies, and the prescription of symptom-control medication. Nonetheless, there is a health care gap in the provision of additional therapies that could complement the pharmacological treatment for FMS management. For this reason, the proposed program combines health education, physical exercise and CBT. It covers topics such as the neurophysiology and pharmacology of pain, postural hygiene, nutrition, insomnia management, memory, sexuality, breathing and relaxation techniques, stretching, coordination exercises and pain, attention, and emotional management. Participants were encouraged to practice what they learned during the sessions, such as walking for 6 to 20 min, at home. However, private practice was not formally registered. The healthcare team comprised female staff including a general practitioner, a physiotherapist, a psychologist, and each health center’s head nurse who delivered the sessions in the primary care centers. Further details of the intervention program can be found in the study protocols [37,40] and Appendix B.

### 2.3. Population and Recruitment

The study sample includes 5 out of 11 primary care centers from the GTTE, south Catalonia, Spain. The MCI groups included in the sample consisted of between 9 and 18 participants. The informers were recruited through purposive heterogeneous sampling to achieve maximum discursive variability based on sociodemographic features (health center, age, sex, birth country, educational level, occupational situation, occupational class, and working status) obtained from the regional digital medical history system for primary care (eCAP).

**Figure 1:**
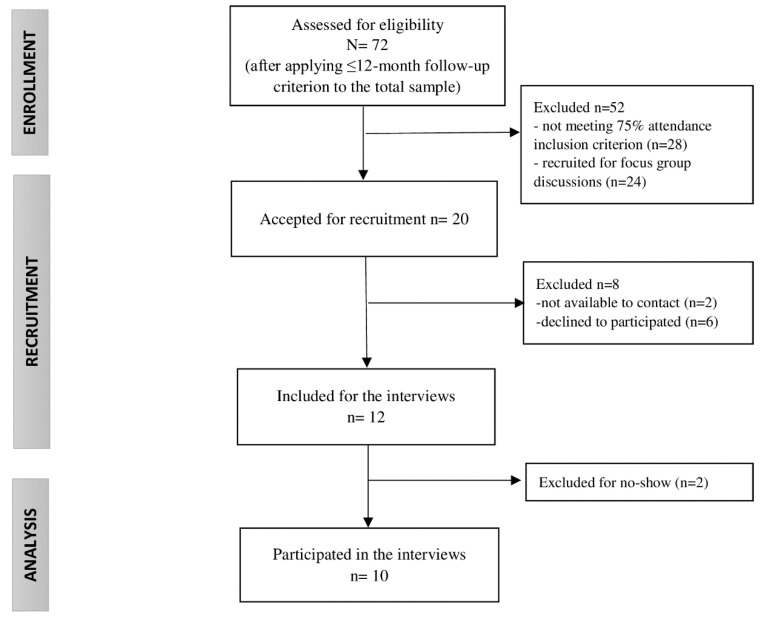
Study sample flow chart.

All participants of the MCI program had a clinical diagnosis of FMS (International Classification of Diseases-10 codes: M79.0, M79.7), were adults, had oral and writing skills in Spanish and/or Catalan, and had a phone contact number. Additionally, the inclusion criteria to participate in this qualitative study involved a minimum of 75% session attendance to the program (equal to or more than 9 out of 12 sessions), up to 12 months since receiving the MCI to avoid memory bias, voluntary participation in the study, and written informed consent. Accordingly, the recruitment process and the sample reached were impacted by informers’ availability within the study, as described in Figure 1.

The recruitment was carried out via phone calls in June 2020, one week earlier than the interview. In order to prevent absenteeism, a reminder text message was delivered the previous day of the meeting. In addition, participants were provided with relevant information about the study’s aim, the interview, and the data protection protocol.

### 2.4. Data Collection

A total of 10 in-depth interviews were conducted to explore patients’ experiences during the MCI program with a mean duration of 30 min

The interviews were scheduled according to participants’ availability. Four of them were conducted face-to-face in the same primary care center (*CAP Baix Ebre de Tortosa*) and six were conducted over telephone due to the COVID-19 pandemic recommendations. The first author (V.M.A.) was the sole interviewer and had no previous contact with the informers before the recruitment process. Only interviewer and interviewee were present during the discussion. The interviewer has an academic background in clinical psychotherapy (BMT) and public health (MPH), with previous experience in qualitative methods, and is currently working towards a PhD in biomedical research.

The interviews were audio-recorded, with prior signed informed consent. A semi-structured discussion schedule including open-ended and follow-up questions (Appendix A) was used, covering the following topics: introduction about the study aims and the interviewer, informer’s overall opinion about the MCI, perceived benefits, implementation of coping strategies in daily life, psychological and emotional perceived changes, social feedback, improvable aspects of the program, and additional comments and suggestions. Interview techniques such as summarizing and clarification were implemented when necessary to facilitate the data collection process during the interviews. Additionally, field notes were made during the meetings by the interviewer that were considered during the data analysis. This discussion schedule was created specifically for this study based on the literature review and previous professional experiences of the research team. Even though it was not previously pilot-tested, it was exhaustively reviewed by the qualitative research team. Additionally, transcripts were not returned to participants for comment and/or correction.

The most illustrative quotations were translated from Catalan and Spanish to English to support our interpretations in the result section.

### 2.5. Data Analysis

A thematic analysis was performed on the informants’ accounts. The hermeneutic phenomenology tenants underlined the analytical process, providing a circular and non-linear interpretative approach which is particularly suitable when exploring subjective lived experiences and including researchers’ preunderstanding [41,42]. Operationally, recordings were transcribed verbatim and carefully read. The researchers’ role was explored for assuring reflexivity and considering possible bias. In this regard, no potential influences were found, and a critical thinking position was adopted to approach the material.

Firstly, preliminary analytical intuitions were registered after a thoughtful reading of the material. Secondly, the text corpus of one of the interviews was analyzed by V.M.A. using NVIVO12 [43] software in order to detect, code, and categorize the most outstanding units of meaning. Consecutively, an analysts’ triangulation process with two research team members (V.M.A. and A.B.) and one external auditor was performed to spot the essential attributes of the patient’s answers, to exchange primary intuitions, and to reach an agreement on the most relevant emerging themes of the interview.

Subsequently, the rest of the analyses of the interviews were conducted based on the previous results and cross-analyzed and compared. After that, a second triangulation with the same researchers was carried out to review and discuss the emerging themes and to address discrepancies through consensus.

Data saturation was assumed once no more themes were captured from the data or more insights were interpreted based on the study objectives. Additionally, the results were double-checked by an external collaborator researcher to guarantee the rigor of the analysis.

## 3. Results

The study sample consists of 10 women, all born in Spain, with a mean age of 58.5 years (min 45, max 73) and a mean of 11 years with a diagnosis of FMS (min 2, max 30). In terms of its impact, the scores obtained at baseline from the Revised Fibromyalgia Impact Questionnaire (FIQR) [44] and the Pain Visual Analogue Scale (PVAS) [45] suggest that the sample was from moderate to severely affected. More details of the sample features are shown in Table 1.

**Figure 2:**
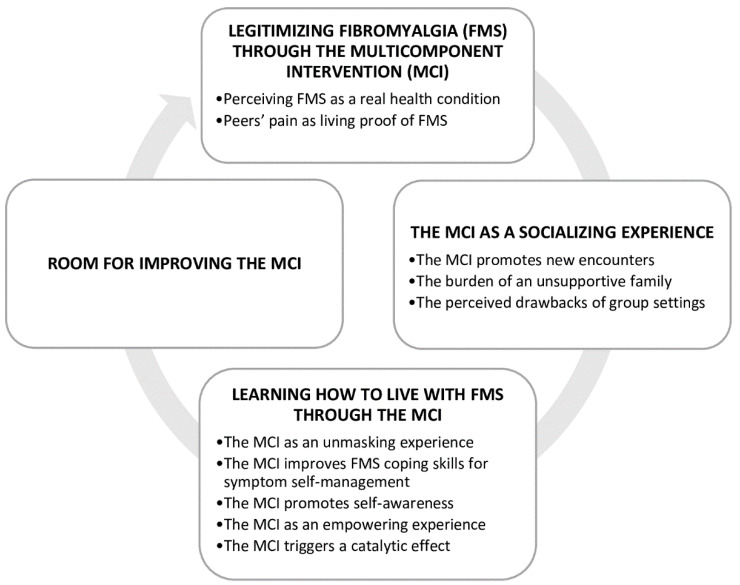
Themes and categories figure.

As a result of the thematic analysis, four themes were identified from the data, as shown in Figure 2.

### 3.1. Theme 1: Legitimizing Fibromyalgia through the MCI

#### 3.1.1. Perceiving Fibromyalgia as a Real Health Condition

According to our informers, the MCI legitimized FMS by validating patients’ illness accounts. Patients reported a sense of being cared about given the provided health education, psychological guidance, professional support, and continuous follow-up. As detected, the invalidation process linked to FMS diagnosis is experienced by patients both physically, due to the lack of medical evidence, and socially, due to the lack of social acknowledgement as a real health issue.

In this regard, informers described hopelessness when their proprioceptive experience is overlooked by the biomedical field and the social network. Further discernment revealed how patients could have undergone emotional dejection, confusion, and doubts regarding their suffering, which led to a nihilistic attitude towards themselves and the healthcare services. While existing treatment strategies are essentially based on pharmacological symptom control and lifestyle changes, patients may not be willing to make any effort if they do not perceive their condition as a real and frequent health problem that requires being addressed to improve functionality and QOL and to prevent future complications. As the following quotes show, interviewees suggested that the proposed MCI contributed to providing clinical value to FMS and its patients, which encouraged them to adopt an active role in their health process:

“The program represents the tranquility of having a point of reference. I used to think it was just my imagination or that I was a complaining person. However, it turns out that it is much more complex than that.”

(P5)

“This experience helped me to show all those people who did not believe me that I was not lying.”

(P9)

#### 3.1.2. Peers’ Pain as Living Proof of FMS

While there is no medical evidence for supporting FMS, it has been detected that peers’ illness experiences provide living testimony to support this health condition. Hence, the significant other seems to play a key role in making sense of one’s health experience and promoting the sense of belonging to a group. Indeed, this biomedical identification process may have facilitated FMS legitimization in the explored sample. We discovered throughout the informers’ accounts that when the different spheres of signification or influence in which they live (social network, legal system, academia, religion, and the media) fail to provide meaning to their health experience, social identity groups offer a socially constructed answer and a sense of being understood. The quote below explains how the patient emotionally struggles when her health experience conflicts with the social and medical perception of FMS, and how the MCI program contributed to it be apprehensible in a context of social validation:

“As this disease is not evident externally, only the person who has it can understand it. It affects you to the point that you cannot move, you do not want to talk, you do not even want to think. It has killed me, indeed. It is very frustrating as it is not reflected anywhere, neither in an analysis, resonance, or image. However, discussing with people who are going through the same thing, make you feel not so weird and shamed. Because this disease is incomprehensible. The program has helped me understand that I am not the only person who is going through it.”

(P3)

### 3.2. Theme 2: The MCI as a Socialising Experience

#### 3.2.1. The MCI Promotes New Encounters

Our interviewees acknowledged the benefits of the group setting leading to new encounters, identification processes, and social networks. Isolation was reported as one of the main consequences of FMS. As informed, patients tend to avoid social contact in order to save explanations on their health progression, changes in their physical performance, and feelings of shame and guilt. The group modality of the MCI offered them the opportunity to create new social networking with people diagnosed alike and who were facing similar challenges. In this regard, finding support from peers emerged as a psychological facilitator for the informers. One of the major strengths of the program transcended the sessions’ setting:

“A very warm bond was born between the participants of the program. Having the opportunity to talk, exchange experiences, and obtain new information blessed me with relief.”

(P6)

“We have made very good friendships with people who suffer the same condition, and we are very fond of each other. We have had phone calls; we have met; we encourage one another daily. We know that we are there for each other and that we are not alone. Finally, someone understands and cares about us.”

(P9)

Additionally, the program has contributed beyond the intra-group relationships. The group effect was found to benefit patients’ self-confidence in social contexts as well as their social initiative. Analytically, as peers become significant others, patients’ self-worth grows and their social performance anxiety and low social perception decrease. Consequently, informers recovered the strength to re-connect with their social environment, which no longer seemed as frightening as before:

“Socially, this experience (the MCI program) has also been beneficial since I no longer feel that I need to justify myself when I cannot do something. Now I relate with others differently because I feel more secure about my illness.”

(P5)

“The program has also helped us to be more sociable in general.”

(P6)

“Before the intervention, I was closed in on myself. There was a time when I practically did not relate to anyone.”

(P9)

#### 3.2.2. The Burden of an Unsupportive Family

Informers reported being poorly supported by family members, particularly by male partners. Patients revealed how their closest discredited FMS. Their need for regular breaks, rescheduling home and work routines, reducing and adapting physical activities, and avoiding intimacy, among others, were frequently taken with hesitancy and intolerance. Informers described this situation as their biggest burden and requested professional help to address it. As portrayed, experiencing one’s health needs being neglected entails a de-subjectivizing process, which impacts both the patient’s well-being and the relationship involved.

Reflecting on these findings, the group effect could have found its ground in the limited family support, whereas peers fill the gap in social understanding. Moreover, given the high prevalence of FMS among women, patients might find more frequent empathy among female peers, a pattern that could have been reinforced by the fact that the MCI program was delivered entirely by female staff.

The following quotes illustrate how FMS challenges the popular beliefs about health and disease within the household context and confronts patients with their partners' denial:

“The only problem I have is that my partner does not understand it… Let’s see He does understand it, but sometimes he tells me “Well, that’s nothing.” He does not quite assimilate it yet.”

(P4)

“I have had a horrible time with this disease because nobody understood me, nobody knew what it was. My husband, for instance, is a person who has never understood it and does not want to; he does not want to believe that I am sick. Since he has cancer, my pain is irrelevant compared to his. And he cannot accept that I am not who I was anymore.”

(P9)

#### 3.2.3. The Perceived Drawbacks of Group Settings

On the other hand, the group setting was also found to be a barrier to personal change. One of the participants who hid behind the group as a shield and due to feeling overwhelmed by other members’ experiences disclosed:

“In the groups, for example, I always take the role of the funny guy. It is my shield. I am not that expressive. However, this experience was helpful to find out that there are many people in worse conditions than me. In this sense, I realized that I am not handling it as bad as I thought. Anyway, there were too many people in the group; too many problems to share; too many mouths to talk; too many thoughts to be said. It is complicated.”

(P1)

Likewise, social comparison processes were detected throughout the informers’ narratives, with a downward contrast. Informers reported gaining perspectives on their health situations in light of hearing about peers in a worse condition. In this line, a feeling of pity towards those peers emerged from their narratives, which helps them to cope with moments of discomfort:

“My mood has improved since I had the opportunity to interact with people who experience the same condition as me or even more seriously. I feel sorry for them.”

(P4)

“Many times, you feel bad, but maybe next to you there is another person who is feeling worse than you. And then you think: ‘well, maybe my situation is not so terrible’. And this strengthens you and gives you a little more encouragement.”

(P8)

### 3.3. Theme 3: Learning How to Live with FMS through the MCI

#### 3.3.1. The MCI as an Unmasking Experience

Participants’ accounts indicated that the MCI facilitated an unmasking and unveiling of their suffering for both themselves and others, a bridge between appearance and reality, and the promotion of understanding and self-acceptance. FMS is an invisible condition that confronts patients’ illness experiences against its social perception. However, supporting FMS legitimacy encouraged patients to open up about their vulnerability, limits, health needs, and emotions. As inferred, learning how to live with FMS includes accepting the condition and facing it beyond social roles and expectations:

“‘Have they called you again?’ He asked, surprised. Since I have to act as Mrs. Strength, it looks like I am not that wounded. Right? But what the program does is show my suffering face. To give it a face […]. It is useful to make the rest of the world realize that something is happening to you. Now I can prove that something else is going on here. From my viewpoint, the experience of the program has been very positive.”

(P1)

“For many years I had had a terrible time with this disease. I did not talk to people. I used to not say what hurt me because everyone could be in pain except me. So, I learned not to say anything. But then, being surrounded by people in the same situation, opened the doors to a world where there were people like me.”

(P9)

#### 3.3.2. The MCI Improves FMS Coping Skills for Symptom Self-Management

Informers revealed having developed new coping skills and a healthier attitude toward FMS, which help them to overcome symptoms, stigma, and uncertainty about the prognosis. Patients gaining insight into their health processes were highlighted as part of the educational benefits of the MCI. In this vein, learning about FMS and coping techniques was highly regarded for symptom self-management. Reflectively, even though widespread pain seems to be still a challenge, adopting new perspectives in FMS may have moved patients beyond the persistent symptomatology and toward acceptance and change. Informers remarked having reduced medication intake; controlled emotional distress; implemented new coping strategies such as physical activity, nutrition, relaxation, and anxiety-control techniques; and adapted their daily activities and exercises to their capabilities. Furthermore, informers stressed the importance of daily exercising; maintaining a routine; following psychological and physical guidelines; or simply but not less important, giving patients something to engage in:

“If I was able to quit the anxiolytics and reduce the panic attacks, it means that the program works.”

(P4)

“I was diagnosed when I was thirty years old, and I practically had to give up my entire life. I lost many friends along the way, and it has been very hard. In the program, I learned how to live with Fibromyalgia. Even though I still have pain, I don’t break down or cry like I used to anymore.”

(P9)

“I notice that even though the exercises we learned with the physiotherapist are quite simple, they make a difference if practicing regularly […]. In this sense, it has been very helpful to have received guidance to know what is particularly useful for us. The nutritional session was also interesting. I have noticed that when eating healthy, I have less muscular pain. Designing a schedule with the psychologist was also very helpful in my view since it encouraged me to keep going and have better track of my progress.”

(P5)

#### 3.3.3. The MCI Promotes Self-Awareness

The MCI successfully promoted participants’ self-awareness, allowing them to acknowledge their physical and psychological needs. As understood, gaining insights into their health condition could have helped the participants to assess their symptoms and possibilities more accurately, to avoid under or overestimating their ailment, and to prioritize themselves. The following quote depicts to what extent FMS patients can experience bodily dissociation as a mechanism to cope with physical pain:

“The program promotes awakening consciousness. For instance, I was not aware that I had so little resistance training capacity […]. Or sometimes, I did not even notice the tachycardias. I have also noticed that my sight is getting worse. Now I understand that I need more time for myself. I remember once when I went for a nail job, and the manicurist did not stop hitting my hands. I was not understanding why he was doing that until I realized that he just wanted me to relax my hands. I was completely unaware of the amount of tension in my hands.”

(P1)

“Thanks to the program, I have incorporated self-awareness. Particularly, I have learned to focus on positive aspects. It has been a valuable experience to me.”

(P2)

Furthermore, reflecting on the informers’ narratives revealed an improvement in their subjective embodiment, which entails connecting their symptoms to their emotional status. Approaching FMS as a complex chronic condition emerged as a key to symptom self-management. In this regard, the proposed MCI aims to address patients suffering holistically including physical, psychological, and social well-being. The metaphor of the fish chasing its tail described in the quote below illustrates the complexity that FMS patients face daily:

“It is the story of the fish chasing its tail. Although we have learned physical techniques, our pain is also related to our emotions. Because in the end, the body retains everything. It is mainly Psychological. In my case, I have an unsolved (psychological) knot. But it refuses to be solved, and I can prove it with my weight raise.”

(P1)

#### 3.3.4. The MCI as an Empowering Experience

Informers’ accounts revealed that the intervention program helped them to increase their autonomy by promoting physical and psychological independence. As experienced by the participants, FMS limits their basic daily activities regarding going out or commuting due to fear of physical impediments and social judgment. Recovering the strength and the courage to face daily life has been interpreted as a valuable experience:

“The MCI encouraged me to be more independent. Before participating in the program, I could not go out alone since I was afraid of falling due to vertigo. I used to panic outdoors. But during the program, I discovered that I was not afraid of falling but of what people could think when they saw me walking with instability. I was ashamed of myself and felt limited. Now, I go everywhere by myself; I no longer care what others think or say.”

(P4)

“I live 12 km from the town where the program took place, and instead of asking my husband to drive me, I preferred to take the bus by myself. Despite my limitations, I used to get on and off the bus following the professionals’ advice about being proactive and increasing physical activity.”

(P7)

#### 3.3.5. The MCI Triggers a Catalytic Effect

FMS was described as a life disruptor that leads to patients abandoning their life projects and losing enthusiasm. In this regard, the MCI program proved to provide patients with a sense of purpose and motivation to carry out new plans. Informers shared their experiences on taking new courses, keeping busy, and exercising daily:

“I was feeling overwhelmed because I could not do anything. But one day the psychologist asked me what I would like to do. And I explained to her that I have always liked languages, but life circumstances had not been in my favor. Anyway, she encouraged me to try it out. Now I study English at a local academy, and I am doing great.”

(P4)

“The professionals encouraged us to keep our minds occupied with other things. For instance, I have signed up for an online course. Being partially disabled and not working, I have more free time to do other things.”

(P5)

### 3.4. Theme 4. Room for Improving the MCI

Informers expressed satisfaction with the experience of the program as a whole and willingness to recommend it. However, they suggested some key points for improvement including more psychological guidance, expressive group therapy, and inviting family members to the sessions to help them to understand the diagnosis and its consequences:

“I would have needed more psychological guidance and strategies. Especially to improve attention and relaxation. For instance, I practise creative meditation through drawing and music in my spare time. It would be interesting to include something like this in the program […]. Besides, some people also needed to talk about family and personal problems because they probably have no other place to do so.”

(P1)

“The staff insisted that the program was not a psychotherapy group. That was crystal clear. But it would have been nice to have a little space for sharing worries in life. They cut us short in this sense.”

(P2)

“The professionals had so little time that they had to explain everything very quickly, and there was no time for letting us express a little more about how we felt about the topic that was being discussed.”

(P3)

“I need someone to explain to my husband, to make him understand what I have because he doesn’t. That’s why it would help if at least one day we could invite our couples to the session. I would sign up right away for that because this is my biggest burden. I would add a bit of therapy so that people could express themselves. what I missed is exactly this, to be able to talk a little more between us.”

(P4)

## 4. Discussion

In this qualitative study, four themes emerged when analyzing FMS patients’ experiences during the MCI program: legitimizing fibromyalgia through the MCI, the MCI as a socializing experience, learning how to live with FMS through the MCI, and room for improving the MCI. Overall, the MCI program was valued unanimously as a positive and insightful experience that should continue as part of the UCC. Another common denominator was the benefits of the social encounter with people with the same diagnosis who could truly understand their suffering without judging them.

### 4.1. The Contributions of Social Support in Legitimizing FMS

Our results provide insights into the role of social support in legitimizing FMS. As Cooper S. and Gilvert L. [46] have disclosed, family, partners, and peers can contribute to accepting the diagnosis, coping with its symptoms and demands, and seeking professional healthcare. In line with this, a meta-synthesis of qualitative studies emphasized the importance of legitimacy in the subjective experience of FMS [21]. Informers reported that only people who suffer from FMS understand it, remarking on the general lack of comprehension of relatives and healthcare professionals. A recent study about the experience of healthcare services for FMS in Sweden indicated that more than half of the sample felt misunderstood by the personnel and were missing an adequate treatment strategy [47].

Our informers showed a tendency to compare themselves with their peers. According to Festinger’s social comparison theory (SC) [48], humans have the drive to compare themselves with others in two different directions: upward and downward by identification or contrast. The former is when people look for similarities with others above them regarding social status or abilities to feel part of that exclusive group (upward identification). On the contrary, they can also find differences with the elite (upward contrast). The latter happens when people compare themselves with others in a worse condition. If they keep their distance from this group, they feel less frustrated with their own (downward contrast). Alternatively, they can highlight the resemblances (downward identification). SC is ubiquitous when there is uncertainty and an information gap and is especially frequent among chronic illnesses [49]. Patients look for answers by mirroring people with the same health condition.

Notably, our informers tended to make a downward contrast with their peers, which helped them to relativize their health condition in contrast to others perceived as equals (since they have the same diagnosis) but worse off and, consequently, allegedly different. Even though this phenomenon could temporarily improve self-esteem, it may redefine patients’ perception of well-being and promote remaining in conformity not visualizing their health needs. Hence, if SC is not skillfully monitored by healthcare professionals, the group effect could be iatrogenic. As a double-sided coin, group interventions can bring social support and belonging, facilitating patients’ health processes, but could also be used as a way to resist change by externalizing one’s health needs to others. Accordingly, the evidence shows that frequent SC can trigger destructive emotions and behaviors [50]. Furthermore, stress appraisal, including intolerance of uncertainty, has been proven as a predictor of SC [49,51]. In order to prevent it, Cabrera-Perona [52] suggests promoting upward identification in SC for intervention programs for chronic pain and fibromyalgia patients due to its self-improvement effect. Therefore, we encourage healthcare providers to approach SC in group settings as a target intervention for FMS patients and not just a feature of human social life.

### 4.2. Monitoring Group-Based Interventions for FMS

The proposed MCI proved to be a socializing experience for its participants. Nevertheless, preventing the drawbacks of a group setting is particularly important considering the high prevalence of alexithymia among FMS patients, which is characterized by difficulty in recognizing and expressing feelings, and it is correlated to psychological distress [53,54]. Contrary to our expectations, some informers referred to struggling when communicating emotions and profound thoughts in front of a group even though they reported significant benefits from this setting. Including emotional awareness and expression psychotherapy [55] in the MCI program in addition to CBT could possibly address this concern as well as cover the improvement aspects proposed by the informers, which coincide with our previous study with focus group discussions [38].

### 4.3. Learning How to Live with FMS: Overcoming Health-Related Guilt

According to our results, health-related guilt emerged among the FMS patients’ accounts and lack of illness legitimacy was indicated as the reason behind it. Sebic et al. [56] found through a systematic review that health-related guilt is a frequent psychological factor in chronic illness associated with more pain, functional impairment, and poor psychological performance. Furthermore, studies corroborate that FMS patients present low levels of forgiveness, both in relation to themselves and to others [57,58]. Forgiveness has been shown to positively impact health and well-being by decreasing stress, improving lifestyle, and encouraging social support [58]. The work of Vallejo et al. [58] demonstrates that high self-forgiveness is related to high levels of active coping and acceptance. Our findings are consistent with previous results suggesting that learning how to live with FMS entails overcoming illness-related guilt and embracing self-forgiveness, which should be included as a psychosocial coping strategy for symptom self-management and well-being promotion [57].

### 4.4. Understanding Health-Related Guilt in FMS from a Gender Perspective

From a gender perspective, guilt is women’s historically inherited burden related to the patriarchal social order [59]. Guilt can be described as the mechanism of gender submission by which we learn to fulfil gender role stereotypes. Indeed, the published evidence indicates that guilt is more frequent and intense in women [60,61]. Research on gender inequalities in health [62] has provided examples of how women’s health has been misunderstood, misdiagnosed, and mistreated by following a biased-men-centered concept of health and well-being in medical practice and healthcare services access [63]. Generally, women are taken less seriously regarding pain, and their accounts are described as “emotional” or “psychological” [64]. A fact that our informers described when being diminished by their social network or partner. Accordingly, Samulowitz et al. [12] have identified gender bias in pharmacological pain treatment. Likewise, Valls [14,65] claims that the differential diagnosis of chronic pain and its gender understanding remains unsolved.

On the other hand, this sex bias may also affect men by being underdiagnosed which delays assessing the peculiarities of FMS in this population and its best treatment approach [13,66]. Studies on men’s illness experience with FMS have found some differences with women’s symptomatology [66,67,68]. For instance, Miró and colleagues [66] found that emotional distress and sleep disorders affect cognitive functions differently between sex. Consequently, therapies for reducing emotional distress seem to be more effective in improving attentional function in women than men. Hence, future research should also focus on the latter sex group in order to specifically tailor interventions to their health needs. On this matter, Werner et al. [69] propose that patients’ illness narratives can be lived out in a “storied form” to overcome stereotyped classifications of gender and illness.

The gender bias in medicine and its gap in comprehensively addressing women’s health needs triggers further consequences. For instance, Shahvisi [70] demonstrated that patriarchal medicine contributes to women’s over-representation within alternative treatments limited in scientific evidence. Note that our informers reported complementary therapeutic strategies to be included in the MCI program. Furthermore, patriarchal medicine is linked to sickness absence and delays in returning to work (RTW), which is a serious concern given FMS patients’ high risk of work absence due to sickness [70], employment impact, and resource consumption [5,71,72]. Women who have reported experiencing negative encounters with healthcare professionals, including teams such as indifference, disrespect, unbelief, and incomprehension, have shown high sick-leave rates and low RTW rates compared to men [73]. Moreover, in agreement with the conclusion of a recent review by Ben-Yosef M. et al. [74] “*patients must be encouraged to continue working*”, since evidence suggests that FMS patients who do not leave their jobs show a promising prognosis. In addition, a recent review proposes that healthcare providers support employers with guidance about workplace accommodation [75].

In light of the evidence, healthcare professionals and intervention programs should contemplate patients’ subjective reports about their illness experiences and circumstances from a psychological and gender approach [64,76]. In this line, the proposed MCI program could address the gender differences in FMS as part of its content considering that it is targeted to both sex groups.

### 4.5. Learning How to Live with FMS: A Mind–Body Approach

The literature recommends that working on a positive body image can improve pain perception [77,78,79]. Indeed, Martinez et al. [80] have found a disturbing embodiment in FMS characterized by poor body awareness. According to our informers’ narratives, the proposed MCI program has helped awaken body needs and sensitivity beyond the pain. Furthermore, Valenzuela-Moguillansky [81] has advanced the hypothesis that disruptions in body awareness led to a paradoxical experience for FMS patients that, while in pain, they cannot actually feel it. An explanation that matches informant P1′s account about not feeling the tension in her hands while receiving a manicure. In order to address these issues, Markey et al. [82] propose that pain acceptance is a key predictor of body image constructs. Thus, healthcare interventions for FMS ought to focus on developing illness coping skills and not just reducing pain levels. Accordingly, a multidisciplinary intervention such as the one assessed would be aimed to regain mobility, sensitivity, and emotional connection with one’s own body in order to re-inhabit it and to overcome the unresponsiveness produced by the experience of pain.

### 4.6. The Multicomponent Intervention Approach in FMS

The proposed MCI program is in line with the latest trend of multidisciplinary approaches for FMS treatment combining pharmacological and non-pharmacological strategies [30]. Even though there is no cure for this condition to date, multicomponent services have been demonstrated to improve patients’ quality of life [83,84]. In this regard, a similar experience conducted in the Royal London Hospital showed that all health outcomes assessed improved significantly at both 6- and 9-month post-MCI-intervention with overall positive feedback from the patients in terms of illness self-management [26]. In accordance with our results, the authors highlighted that, ultimately, the intervention helped patients to learn to deal with FMS. Even though many other therapeutic strategies have been revised including pilates, yoga, mindfulness, walking programs, water exercises, and multimedia techniques, among others, a new systematic review suggests that the most effective treatments are those that incorporate health education and complete physical activity (including aerobic and relaxation exercises) [83]. In addition, the psychological component is also indicated as a key factor in supporting patients’ mental health and developing coping skills, which coincide with our findings.

### 4.7. Strengths and Limitations

Our study follows the consolidated criteria for reporting qualitative research (COREQ) [85] in order to achieve scientific rigor. The sample recruitment was systematically and purposively conducted to guarantee variability and to avoid selection bias. Furthermore, two analytics triangulations were performed during the analysis process involving two experienced members of the qualitative research team and one external auditor.

Methodologically, the reported findings result from a non-linear interpretation process that intends to summarize the “essence” of the informers’ accounts by means of themes that recover the embodied lived experiences in their narratives. In this regard, themes within the context of hermeneutic phenomenology attempt to overcome a superficial description of patterns in the text, which is particularly suitable for modest samples.

Notwithstanding the sample size, efforts were made to obtain quality data and to analyze it with reflexivity and rigor. As a result, this study pretends to understand the impact and benefits of an MCI program on patients’ subjectivity from a critical thinking perspective.

Regarding the limitations, and since the interviewee sample only included those with high assistance records and willingness to participate in the study, the results could have overestimated the benefits of the program. However, the interview schedule included questions concerning improvement aspects for the MCI and interpretations were focused on the unveiled effects of the program.

Moreover, the sample did not include men as the only two possible candidates did not agree to be interviewed. Women’s FMS high prevalence contributed to a limited number of men within the MCI program. Moreover, the data collection process was conducted during the COVID-19 pandemic, which could have affected participation. Future projects should make efforts to recruit this population group. Additionally, the informers included belonged to only 5 out of 11 primary care centers of the health region owing to inclusion criteria requirements and sample availability. Nevertheless, since the MCI was performed the same in every center, we do not consider that this aspect could have impacted the results significantly.

Regarding the data collection, due to the COVID-19 outbreak telephonic interviews had to be conducted in some cases. Therefore, the interviewer–interviewee relationship could have been more impersonal than face-to-face interviews since it was the first contact between the informant and the researcher. However, the interviewer provided relevant information about her background to the participants and encouraged a natural and fluent conversation, including follow-up questions and promoting meaningful exchanges, in order to reduce this potential limitation.

In addition, even though the results were not delivered to the informers for review and feedback, an outside research group was consulted for further validation.

Lastly, our findings may not be generalizable beyond the study sample. Nevertheless, the MCI program could be tailored and assessed in other contexts.

## 5. Conclusions

Our study has contributed to gaining novel insight into FMS patients’ lived experiences during an MCI in primary care. Informers agreed on the program as a revealing, insightful, and motivating experience that has facilitated illness knowledge and acceptance and improved their coping skills and self-management in daily life. Furthermore, peer support was emphasized as a key benefit, beyond clinical outcomes, due to the sense of comprehension, bonding, and encouragement that remains active post-intervention as a positive social network. Gender awareness and empowerment have also been observed as remarkable results. In this regard, including gender diversity within the staff might help patients to deliver information to their partners. Moreover, complex psychological mechanisms were identified intra-group level, which the professionals should skillfully monitor and intervene. In addition, the inclusion of expressive psychological group therapy and inviting relatives to the sessions were claimed as improvement aspects of the program. Nonetheless, our findings warrant further discussion. Finally, these results will allow for adjusting the program to patients’ health needs to broaden its benefits and to support the UCC.

## Figures and Tables

**Table 1:** Sample description.

ID	Interview Type	Health Area *	Age (Years)	Civil Status	Occupational Situation	Educational Level	Occupational Class **	Years Since Diagnosis	Months after MCI	Session Attendance (%)	FIQR	PVAS
P1	face-to-face	1	46	married	working	secondary	IVa	7	12	83%	47.33	8
P2	phone	2	73	married	retired	university	I	9	5	100%	65	10
P3	phone	3	59	divorced	working	primary	IVb	2	12	92%	88.67	8
P4	face-to-face	1	51	married	unemployed	primary	IVb	13	12	75%	78.17	8
P5	phone	1	54	divorced	disabled	secondary	IVa	11	6	83%	96.33	8
P6	phone	2	45	married	unemployed	secondary	II	2	5	75%	42.17	7
P7	phone	1	71	married	retired	secondary	IVb	30	6	75%	81.17	8
P8	face-to-face	1	65	married	working	primary	IVa	12	6	92%	64.67	8
P9	phone	4	63	married	retired	primary	IVb	22	12	83%	93.17	6
P10	face-to-face	1	58	married	retired	primary	IVb	2	6	75%	69.33	5

MCI = Multicomponent intervention; FIQR = Revised Fibromyalgia Impact Questionnaire; PVAS = Pain Visual Analogue Scale. * Health area: 1 = Tortosa; 2 = La Rápita; 3 = Flix; 4 = Aldea. ** Occupational class: I (professionals); II (intermediate occupations); IVa (skilled manual workers); IVb (other manual workers).

## Data Availability

Due to ethical restrictions, supporting data cannot be made openly available. Participants of this study did not agree for their data to be shared publicly since it may contain potentially identifying or sensitive patient information. Nonetheless, specific data analysis results, such as a section of the audit trail, could be shared by the corresponding author, V.M.A., upon reasonable request and are subjected to a non-disclosure agreement.

## Appendix A

### Discussion Schedule

1. What is your general opinion about the fibromyalgia program in which you participated? How would you describe the experience?
2. Could you mention any benefit you may have perceived from the program if any?
3. Have you implemented any of the techniques and learnings performed during the sessions in your daily life? Which ones? How often?
4. Do you think that this experience has helped you to relate differently to your body? How so?
5. How do you feel emotionally after participating in this program? Could you describe it (a bit more)?
6. To what extent the intervention has improved your fibromyalgia coping strategies? How so?
7. To what extent has this healthcare experience developed your social skills? How so?
8. What kind of feedback have you received from people close to you who have experienced your process before and after the intervention?
9. What would you change or improve in the program?
10. Why would you recommend this intervention program to other patients with a diagnosis of fibromyalgia if so?
11. Would you like to comment on something else or address any other issue that has not been discussed?

## Appendix B

**Table A1:** Multicomponent intervention description.

Sessions	Health Education Component	Physical Component	Psychological Component: Cognitive Behavioral Therapy (CBT)
1	Introduction to Multicomponent Therapy (30 min)	6 min walking test (45 min)	Cognitive-behavioral strategies (45 min)
2	Neurophysiology and Pharmacology of Pain (1 h)		Breathing and Relaxation (1 h)
3		Breathing and Relaxation (1 h)	Management of Attention (1 h)
4	Techniques of Postural Hygiene (1 h)	Stretching exercises (1 h)	
5	Nutrition (1 h)	Breathing and Relaxation (1 h)	
6	Management of Insomnia (1 h)	Joint exercises/games (1 h)	
7	Memory (1 h)	Stretching/joint exercises (1 h)	
8	Sexuality (1 h)	Postural hygiene/stretching (1 h)	
9		Strength/joint exercises (1 h)	Activities and Mood (1 h)
10		Coordination exercises (1 h)	Planning pleasant activities (1 h)
11		Breathing and Relaxation (1 h)	Management of Difficulties (1 h)
12		6 min walking test (1 h)	Identification of Objectives (1 h)
Total time	6.5 h	10.75 h	6.75 h
